# Supplementary material for: A Novel Quadruple Gene-Deleted BoHV-1-Vectored RVFV Subunit Vaccine Induces Humoral and Cell-Mediated Immune Response against Rift Valley Fever in Calves
Source: Viruses. 2023 Oct 30;15(11):2183. doi: 10.3390/v15112183 (PMC10674938; doi:10.3390/v15112183)
Supplement: Supplementary file 1 [file viruses-15-02183-s001.zip › viruses-2667485-supplementary.pdf]

5′ **GGTACC**TAGTTATTAATAGTAATCAATTACGGGGTCATTAGTTCATAGCCCATATATGGAGTTCCGCGT

Kpn-1 CMV-IE promoter

TACATAACTTACGGTAAATGGCCCGCCTGGCTGACCGCCCAACGACCCCCGCCCATTGACGTCAATAATG
ACGTATGTTCCCATAGTAACGCCAATAGGGACTTTCCATTGACGTCAATGGGTGGAGTATTTACGGTAAA
CTGCCCACTTGGCAGTACATCAAGTGTATCATATGCCAAGTACGCCCCCTATTGACGTCAATGACGGTAA
ATGGCCCGCCTGGCATTATGCCCAGTACATGACCTTATGGGACTTTCCTACTTGGCAGTACATCTACGTA
TTAGTCATCGCTATTACCATGGTGATGCGGTTTTGGCAGTACATCAATGGGCGTGGATAGCGGTTTGACT
CACGGGGATTTCCAAGTCTCCACCCCATTGACGTCAATGGGAGTTTGTTTTGGCACCAAAATCAACGGGA
CTTTCCAAAATGTCGTAACAACTCCGCCCCATTGACGCAAATGGGCGGTAGGCGTGTACGGTGGGAGGTC
TATATAAGCAGAGCTGGTTTAGTGAACCGTCAGATCCGCTAGCGCTACCGGT**GCCGCCACC**MQGPTLAVL

Kozak sequence BoHV-1 gD signal sequence (1-19 aa)

GALLAVAVSL**M**EDPHLRNRPGKGHNYIDGMTQEDATCKPVTYAGACSSFDVLLEKGKFPLFQSYAHHRTL

RVFV Gn sequence (1, 154-581 aa)

LEAVHDTIIAKADPPSCDLLSAHGNPCMKEKLVMKTHCPNDYQSAHYLNNDGKMASVKCPPKYELTEDCN
FCRQMTGASLKKGSYPLQDLFCQSSEDDGSKLKTKMKGVCEVGVQALKKCDGQLSTAHEVVPFAVFKNSK
KVYLDKLDLKTEENLLPDSFVCFEHKGQYKGTMDSGQTKRELKSFDISQCPKIGGHGSKKCTGDAAFCSA
YECTAQYANAYCSHANGSGIVQIQVSGVWKKPLCVGYERVVVKRELSAKPIQRVEPCTTCITKCEPHGLV
VRSTGFKISSAVACASGVCVTGSQSPSTEITLKYPGISQSSGGDIGVHMAHDDQSVSSKIVAHCPPQDPC
LVHDCIVCAHGLINYQCHTRVLKCLKIAPRKVLNPLMWITAFIRWIYKKMVARVADNINQVNREIGWMEG

Gn cytoplasmic tail (605-674 aa)

GQLVLGNPAPIPRHAPIPRMAPTRPPNTATRPWQHVDAIKEALSLLNHSSDTDAVMNDTEVVSEKFDSQE

Bovine GMCSF (1, 18-143 aa)

PTCLQTRLKLYKNGLQGSLTSLMGSLTMMATHYEKHCPPTPETSCGTQFISFKNFKEDLKEFLFIIPFDC
WEPAQK**GACTACAAAGACGATGACGACAAG**GSGATNFSLLKQAGDVEENPGP**M**CSELIQASSRITTCSTE

Flag Tag GSG + Peptide 2A RVFV Gc with transmembrane and cytoplasmic (1, 691-1197 aa)

GVNTKCRLSGTALIRAGSVGAEACLMLKGVKEDQTKFLKIKTVSSELSCREGQSYWTGSFSPKCLSSRRC
HLVGECHVNRCLSWRDNETSAEFSFVGESTTMRENKCFEQCGGWGCGCFNVNPSCLFVHTYLQSVRKEAL
RVFNCIDWVHKLTLEITDFDGSVSTIDLGASSSRFTNWGSVSLSLDAEGISGSNSFSFIESPGKGYAIVD
EPFSEIPRQGFLGEIRCNSESSVLSAHESCLRAPNLISYKPMIDQLECTTNLIDPFVVFERGSLPQTRND
KTFAASKGNRGVQAFSKGSVQADLTLMFDNFEVDFVGAAVSCDAAFLNLTGCYSCNAGARVCLSITSTGT
GSLSAHNKDGSLHIVLPSENGTKDQCQILHFTVPEVEEEFMYSCDGDERPLLVKGTLIAIDPFDDRREAG
GESTVVNPKSGSWNFFDWFSGLMSWFGGPLKTILLICLYVALSIGLFFLLIYLGRTGLSKMWLAATKKAS
ggtaagcctatccctaaccctctcctcggtctcgattctacgcgtaccggt**TAA***CTGATCATAATCAGCC*

V5 Epitope Stop codon SV40 PolyA

ATACCACATTTGTAGAGGTTTTACTTGCTTTAAAAAACCTCCCACACCTCCCCCTGAACCTGAAACATAA
AATGAATGCAATTGTTGTTGTTAACTTGTTTATTGCAGCTTATAATGGTTACAAATAAAGCAATAGCATC
ACAAATTTCACAAATAAAGCATTTTTTTCACTGCATTCTAGTTGTGGTTTGTCCAAACTCATCAATGTAT
*CTTA***AAGCTT**-3′

Hind III

Figure S1: RVFV Gn-FLAG-P2A-Gc-V5 chimera sequence. RVFV Gn and Gc sequence with the KpnI and HindIII sites were designed to include 5’-3’ the following: Kpn1 site, CMV-IE promoter, Kozak sequence, BoHV-1 gD signal peptide, RVFV Gn without the transmembrane domain, Gn cytoplasmic tail, Bovine GMCSF, Flag tag, GSG sequence to improve cleavage efficiency, Peptide 2 A sequence, RVFV Gc region with transmembrane domain, V5 epitope, stop codon, SV polyA tail sequence and HindIII site.

Table S1. Vaccine virus or vector virus isolation from nasal swab samples in MDBK cells.

S no.:	Group	Animal #	Nasal virus shedding following immunization (Plaque forming units/nasal swab) (Days post-vaccination)								
			Day 0	Day 3	Day 5	Day 7	Day 14	Day 21	Day 28	Day 28	Day 33
1	BoHV-1qmv vector control group	868	0	3.0×10^3	2.5×10^2	5.0×10^1	0	0	0	0	0
2		866	0	2.0×10^2	0	5.0×10^1	0	0	0	0	0
3		890	0	2.0×10^2	0	0	0	0	0	0	0
1	BoHV-1qmv Sub-RVFV vaccine group	865	0	4.8×10^3	0	0	0	0	0	0	0
2		860	0	4.0×10^2	0	0	0	0	0	0	0
3		875	0	5.0×10^1	5.0×10^1	0	0	0	0	0	0
4		862	0	2.0×10^2	0	0	0	0	0	0	0
5		867	0	5.0×10^2	0	0	0	0	0	0	0
6		876	0	3.0×10^3	0	0	0	0	0	0	0
7		879	0	9.0×10^2	0	0	0	0	0	0	0
8		828	0	4.3×10^3	0	0	0	0	0	0	0

Table S2. Quantification of bovine herpesvirus type 1 (BoHV-1) genome copies in nasal swab samples collected from the vaccine virus or vector virus inoculated calves by qPCR.

S no.:	Group	Animal #	Nasal virus shedding following immunization (qPCR; viral genome copies/nasal swab) (Days post-vaccination)								
			Day 0	Day 3	Day 5	Day 7	Day 14	Day 21	Day 28	Day 28	Day 33
1	BoHV-1qmv vector control group	868	0	138,585	144,680	259	0	0	0	0	0
2		866	0	1900	1015	0	0	0	0	0	0
3		890	0	17,694	1541	0	0	0	0	0	0
		Average	0	52,726	49,078	86	0	0	0	0	0
1	BoHV-1qmv Sub-RVFV vaccine group	865	0	241,080	10,871	0	0	0	0	0	0
2		860	0	10,032	13,810	0	0	0	0	0	0
3		875	0	5913	111,590	0	0	0	0	0	0
4		862	0	2868	945	0	0	0	0	0	0
5		867	0	5673	200,640	0	0	0	0	0	0
6		876	0	48,049	12,677	57	0	0	0	0	0
7		879	0	22,754	27,315	0	0	0	0	0	0
8		828	0	35,225	5859	77	0	0	0	0	0
		Average	0	46,449	47,963	16	0	0	0	0	0

Table S3. Bovine herpesvirus type 1 (BoHV-1)-specific neutralizing antibody titer in serum.

S no.:	Group	Animal #	BoHV-1-specific neutralizing antibody titer in serum (Days post-vaccination)					
			Day 0	Day 7	Day 14	Day 21	Day 28	Day 33
1	BoHV-1qmv vector control group	868	<4	17.57	46.11	83.05	66.64	*
2		866	<4	11.59	39.27	53.66	20.92	16.40
3		890	<4	15.78	43.39	55.17	26.6	20.97
		Average	<4	14.98	42.92	63.96	38.05	18.66
1	BoHV-1qmv Sub-RVFV vaccine group	865	<4	18.84	61.37	94.35	28.62	*
2		860	<4	22.12	70.45	103.6	41.63	39.62
3		875	<4	<4	5.67	13.12	12.5	*
4		862	<4	7.43	8.80	16.53	10.91	9.94
5		867	<4	11.63	20.96	25.01	15.44	10.19
6		876	<4	7.42	9.03	14.73	11.61	7.93
7		879	<4	4.11	5.26	11.40	8.15	*
8		828	<4	<4	6.02	11.07	10.04	*
		Average	<4	11.93	23.44	36.23	17.36	16.92

*** - Sacrificed on day 28 post vaccination**

Table S4. Rift Valley fever virus (RVFV)-specific neutralizing antibody titer in serum.

S no.:	Group	Animal #	RVFV-specific neutralizing antibody titer in serum (Days post-vaccination)					
			Day 0	Day 7	Day 14	Day 21	Day 28	Day 33
1	BoHV-1qmv vector control group	868	<4	<4	<4	<4	<4	*
2		866	<4	<4	<4	<4	<4	<4
3		890	<4	<4	<4	<4	<4	<4
		**Average**	**<4**	**<4**	**<4**	**<4**	**<4**	**<4**
1	BoHV-1qmv Sub-RVFV vaccine group	865	<4	10.02	13.13	54.71	53.98	*
2		860	<4	9.87	11.39	26.45	14.19	9.75
3		875	<4	4.09	5.36	13.02	10.34	*
4		862	<4	4.06	5.64	5.93	9.42	9.00
5		867	<4	5.79	6.49	8.17	7.67	6.29
6		876	<4	5.54	6.3	7.77	12.02	32.72
7		879	<4	5.66	6.48	10.95	15.67	*
8		828	<4	6.64	7.61	10.95	7.57	*
		**Average**	**<4**	**6.46**	**7.68**	**17.24**	**16.34**	**14.44**

*** - Sacrificed on day 28 post vaccination**

Table S5. Fold change in interferon (IFN)-gamma mRNA expression in peripheral blood mononuclear cells collected from immunized calves upon stimulation with heat-inactivated Rift Valley fever virus antigen.

S no.:	Group	Animal #	Fold change in IFN-gamma mRNA expression after PBMC stimulation (Days post-vaccination)		
			Day 0	Day 7	Day 14
**1**	**BoHV-1qmv vector control group**	**868**	1	1.01	1
**2**		**866**	1	0.87	1.13
**3**		**890**	1	1	0.9
		**Average**	**1**	**0.96**	**1.01**
**1**	**BoHV-1qmv Sub-RVFV vaccine group**	**865**	0.97	15.6	7.5
**2**		**860**	1	8.6	2
**3**		**875**	1	11.2	3.5
**4**		**862**	1	4.6	3.7
**5**		**867**	1	16.40	3.31
**6**		**876**	1	5.74	3.46
**7**		**879**	1	2.51	2.03
**8**		**828**	1	2.07	3.55
		**Average**	**1**	**7.30**	**3.08**
